# Deciphering Folate Receptor alphaGene Expression and mRNA Signatures in Ovarian Cancer: Implications for Precision Therapies

**DOI:** 10.3390/ijms252211953

**Published:** 2024-11-07

**Authors:** Maria Kfoury, Pascal Finetti, Emilie Mamessier, François Bertucci, Renaud Sabatier

**Affiliations:** 1 Medical Oncology Department, Institut Paoli-Calmettes, 13009 Marseille, France; 2 Predictive Oncology Laboratory, Inserm UMR1068, Centre National de la Recherche Scientifique (CNRS) UMR7258, Centre de Recherche en Cancérologie de Marseille (CRCM), Institut Paoli-Calmettes, Aix-Marseille University U105, 13009 Marseille, France

**Keywords:** ovarian carcinoma, folate receptor alpha, gene expression, mRNA

## Abstract

Antibody–drug conjugates targeting folate receptor alpha (FRα) are a promising treatment for platinum-resistant ovarian cancer (OC) with high FRα expression. Challenges persist in accurately assessing FRα expression levels. Our study aimed to better elucidate FRα gene expression and identify mRNA signatures in OC. We pooled OC gene expression data from 16 public datasets, encompassing 1832 OC and 30 normal ovarian tissues. Additional data included DNA copy number and methylation data from TCGA and protein data from 363 cancer cell lines from the Broad Institute Cancer Cell Line Encyclopedia. *FOLR1* mRNA expression was significantly correlated with protein expression in pan-cancer cell lines and ovarian cancer cell lines. *FOLR1* expression was higher in OC samples than in normal ovarian tissues (OR = 3.88, *p* = 6.97 × 10^−12^). Patients with high *FOLR1* expression were more likely to be diagnosed with serous histology, FIGO stage III–IV, and high-grade tumors; however, nearly similar percentages of patients with low *FOLR1* expression were also diagnosed with these features. *FOLR1* mRNA expression was not correlated with platinum sensitivity or complete surgery, nor with prognosis. However, we identified a 187-gene signature associated with high *FOLR1* expression that was significantly associated with improved survival (HR = 0.71, *p* = 1.18 × 10^−6^), independently from clinicopathological features. We identified a gene expression signature correlated to high FRα expression and OC prognosis, which may be used to refine therapeutic strategies targeting FRα in OC. These findings warrant validation in larger cohorts.

## 1. Introduction

Ovarian carcinoma (OC) is the deadliest gynecological cancer in Western countries, with an estimated 19,680 new cases and 12,740 OC-related deaths predicted to occur in 2024 in the United States [1]. Despite recent advances with PARP inhibitors, OC prognosis remains poor, as OC becomes platinum-resistant, with a median overall survival of close to 12 months. For the first time, the prognosis of platinum-resistant OC can be improved using mirvetuximab soravtansine, an antibody–drug conjugate that includes a monoclonal antibody targeting folate receptor alpha (FRα) [2]. FRα has thus emerged as a compelling target in OC due to its overexpression in a significant subset of these tumors. Functioning as a glycosylphosphatidylinositol-anchored cell surface protein, the folate receptor plays a pivotal role in transporting folate into cells and is essential for DNA synthesis, repair, and methylation processes [3].

Compared with those in normal tissues, the expression levels of FRα, which is encoded by the *FOLR1* gene, are notably elevated in OC cells [4,5]. This upregulation in almost 80% of OCs is associated with increased tumor proliferation, survival, and resistance to chemotherapy, making it a pivotal player in disease progression and therapeutic resistance [6,7,8]. Remarkably, FRα expression persists after chemotherapy in recurrent and metastatic lesions, reinforcing its importance as a potential therapeutic target [9,10].

In the realm of targeted therapy, mirvetuximab soravtansine, a first-in-class antibody–drug conjugate that targets FRα, has marked a significant milestone in OC treatment. Comprising a FRα-binding antibody, a cleavable linker, and the maytansinoid DM4, a potent tubulin-targeting agent, mirvetuximab soravtansine’s positive results in the single-arm pivotal SORAYA trial in FRα-positive, bevacizumab-pretreated, platinum-resistant, high-grade serous OC led to its accelerated approval by the Food and Drug Administration in 2022 [11]. The benefit in survival was confirmed in MIRASOL, a phase 3 global, confirmatory, open-label, randomized, controlled trial comparing mirvetuximab soravtansine with the investigators’ choice of chemotherapy in platinum-resistant, high-grade serous OC with high FRα tumor expression (≥75% of cells with ≥2+ staining intensity) [2]. This benefit over chemotherapy was also observed with respect to progression-free survival and objective response. Mirvetuximab soravtansine is the first antibody–drug conjugate approved for OC.

However, challenges persist in accurately assessing FRα expression, as evident from the findings of the FORWARD I trial, wherein suboptimal scoring methods diluted the observed treatment effect of mirvetuximab soravtansine [12]. These findings emphasize the critical need for refined evaluation techniques to fully comprehend the therapeutic potential of FRα-targeted therapies.

Understanding FRα gene expression at the messenger RNA (mRNA) level holds significant promise for elucidating the intricacies of its regulation and downstream effects in OC. Investigating the mRNA signatures linked to FRα expression offers a unique opportunity to uncover the molecular mechanisms driving its overexpression, potentially revealing novel therapeutic targets or predictive biomarkers crucial for tailored treatment strategies.

In this study, our primary aim was to comprehensively describe FRα gene expression in OC clinical samples and identify associated mRNA signatures.

## 2. Results

### 2.1. Correlation of FOLR1 mRNA Expression with Protein Expression Levels in Cancer Cell Lines

We first analyzed the gene (RNA-seq) and protein (reverse-phase protein array) expression data of *FOLR1* in 363 cancer cell lines, including 17 ovarian cell lines, from the Cancer Cell Line Encyclopedia (CCLE), publicly hosted on the DepMap portal. *FOLR1* mRNA expression was significantly correlated with protein expression in both cancer cell lines (r = 0.82, *p* = 5.7 × 10^−76^) and ovarian cell lines (r = 0.87, *p* = 1.03 × 10^−5^) (Supplementary Figure S1).

### 2.2. Correlations of FOLR1 mRNA Expression with Clinicopathological Features

We evaluated *FOLR1* mRNA expression in 1832 cancer ovarian samples and 30 normal ovarian samples. The median patients’ age at diagnosis was 59 years (range 21–90). Most patients had serous (N = 929; 92%), advanced-stage (FIGO stage III–IV; N = 1195; 88%), and pathological high-grade (N = 1041; 75%) carcinoma, with a platinum-sensitive profile (N = 1183; 88%) after first-line treatment (Table 1). *TP53* was mutated in 97% of the patients whose data were available, and 22% of the tumors presented a *BRCA1/2* mutation. Application of the CLOVAR classification [13] identified 28.5% of the tumors as differentiated, 22.9% as immunoreactive, 23.8% as mesenchymal, and 24.8% as proliferative.

**Figure 1:**
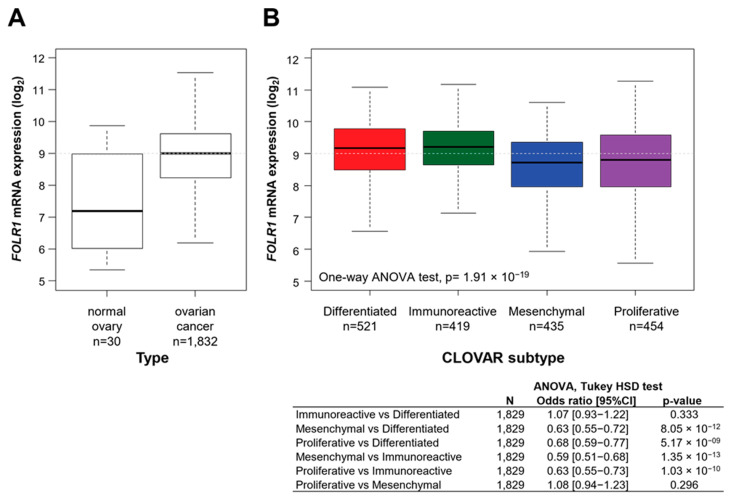
Box plot of *FOLR1* mRNA expression according to (**A**) type of sample and (**B**) CLOVAR molecular subtypes (Red: Differenciated; Green: Immunoreactive; Blue: Mesenchymal; Purple: Proliferative). Significance was assessed by using Student’s t-test and one-way ANOVA, respectively.

The correlations between *FOLR1* mRNA expression and clinicopathological features are shown in Table 1. We observed differences in *FOLR1* expression by histological type, tumor grade and FIGO stage. Patients with high *FOLR1* expression were more likely to be diagnosed with serous histology (OR = 2.45 [1.4946–4. 021]), FIGO stage III–IV tumors (OR = 1.71 [1.21–2.44]), and high-grade tumors (OR = 1.45 [1.12–1.87]). However, nearly similar percentages of patients with low *FOLR1* expression were also diagnosed with these features. No correlation was found with patients’ age, platinum sensitivity, or achievement of complete cytoreductive surgery. Differentiated (OR = 1.32 [1.07–1.62], *p* = 7.60 × 10^−3^) and immunoreactive (OR = 2.47 [1.98–3.07], *p* = 2.10 × 10^−17^) CLOVAR subtypes were associated with high *FOLR1* mRNA expression, whereas mesenchymal and proliferative tumors presented lower *FOLR1* expression (*p* < 0.001) (Figure 1B).

### 2.3. FOLR1 mRNA Expression Is Not Correlated with Survival

Progression-free survival was evaluated in 1207 patients, and overall survival was evaluated in 1470 patients. The median follow-up was 35.8 months (range, 1–243) in the whole population, with a median PFS equal to 35 months (range, 1–243) and median OS equal to 45 months (range, 1–243). No association was found between *FOLR1* mRNA expression and PFS or OS. The median PFS and OS were 34 and 43 months, respectively, in the low *FOLR1* expression group compared with 36 and 47 months, respectively, in the high *FOLR1* expression group. A time-dependent analysis revealed no association between *FOLR1* mRNA expression and survival at 6 months, 12 months, or more than 12 months (Supplementary Figure S2).

**Figure 2:**
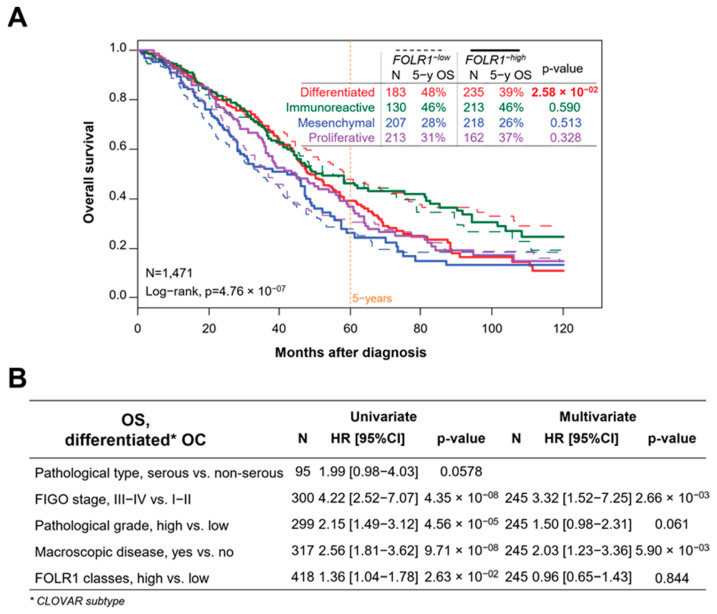
Correlation of *FOLR1* mRNA expression with survival. (**A**) Kaplan–Meier curve for overall survival according to *FOLR1* mRNA expression in CLOVAR molecular subtypes (Red: Differenciated; Green: Immunoreactive; Blue: Mesenchymal; Purple: Proliferative);significance assessed by using Log-rank. (**B**) Uni- and multivariate Cox regression analyses of overall survival in the differentiated CLOVAR subtype according to *FOLR1* mRNA expression and usual clinicopathological features; significance assessed by using Wald’s test.

A statistically significant difference in PFS and OS was observed in the CLOVAR-differentiated subtype (Figure 2A, Supplementary Figure S3A). The five-year PFS rate was 40% in the low *FOLR1* expression group and 29% in the high *FOLR1* expression group (HR = 1.44, 95% CI [1.08–1.91]; *p* = 0.011). The five-year OS rates were 48% and 39%, respectively (HR = 1.36; 95% CI [1.04–1.78]; *p* = 0.026). However, this difference did not remain in multivariate analyses including known prognostic features (pathological subtypes, FIGO stages, grade, and presence of macroscopic residual disease after surgery) (Figure 2B, Supplementary Figure S3B), suggesting the presence of confounding factors in univariate analysis.

### 2.4. FOLR1 mRNA Expression and Associated Biological Processes

**Figure 3:**
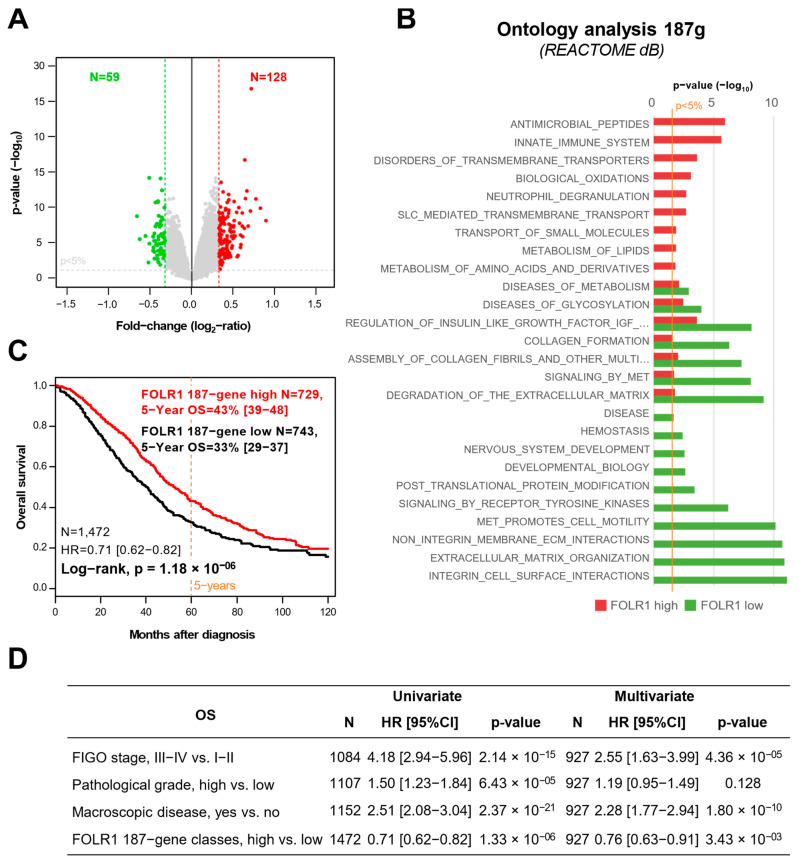
Analysis of *FOLR1* mRNA expression profile. (**A**) Volcano plot representation of differentially expressed genes between *FOLR1* expression groups in 571 TCGA OC (288 FORL1-high vs. 283 -low). The red dots represent the significant genes upregulated in *FOLR1*-high versus -low, and the green dots represent the significant genes downregulated in *FOLR1*-high versus -low. (**B**) Ontology analysis based on the 187 differential genes. All significative pathways are represented. (**C**) Kaplan–Meier curve for overall survival according to the *FOLR1* 187-gene signature. (**D**) Uni- and multivariate analysis of overall survival according to the *FOLR1* 187-gene signature and clinicopathological features. Abbreviations: OS: Overall survival, HR: Hazard Ratio.

To explore the biological alterations that are associated with the *FOLR1* expression status, we applied supervised analysis to the TCGA dataset (N = 571). We identified 187 genes that were differentially expressed between tumors with *FOLR1* upregulation (N = 288) versus tumors without *FOLR1* upregulation (N = 283) (Figure 3A, Supplementary Table S2). Ontology analysis of the 187-gene revealed that genes involved in the innate immune system and transmembrane transport were overexpressed in the “*FOLR1*-high” tumors, whereas genes involved in integrins and extracellular matrix interactions were underexpressed (Figure 3B). The robustness of this gene signature was verified in the training set and, more importantly, in the independent validation set by using a metagene-based prediction score (Supplementary Figure S4).

The *FOLR1* high-like signature was significantly associated with improved overall survival, with a hazard ratio of 0.71 (95% CI [0.62–0.82]; *p* = 1.18 × 10^−6^, log-rank test) (Figure 3C). The five-year OS rates were 43% (95% CI [39–48]) for *FOLR1* high-like tumors and 33% (95% CI [29–37]) for *FOLR1* low-like tumors. Multivariate analysis confirmed that this prognostic impact was independent from clinicopathological features (Figure 3D).

### 2.5. Correlation of FOLR1 mRNA Expression with Members of the Folate Signaling Pathway

We explored the association of *FOLR1* expression with the expression of genes encoding five other molecules involved in the folate/FRα signaling pathway in normal ovary and ovarian cancer tissues: *MTHFR*, methylene tetrahydrofolate reductase; *FOLH1*, glutamate carboxypeptidase; *TYMS*, thymidylate synthase; *DHFR*, dihydrofolate reductase; and *SLC19A1* folate transporter 1. The expression levels of *TYMS*, *DHFR*, and *SLC19A1* were significantly higher in cancer ovarian samples than in normal ovarian samples. No correlation was found between *FOLR1* mRNA expression and the other genes involved in the folate pathway. Only expression of *SLC19A1*, encoding a reduced folate carrier (RFC), was associated with overall survival in univariate analysis (Supplementary Figure S5).

### 2.6. Correlation of mRNA Expression, DNA Methylation, and Copy Number Alterations

Finally, we studied, on the basis of TCGA data, the correlations of mRNA expression with promoter methylation and copy number alterations in the six abovementioned genes involved in the folate signaling pathway. Copy gain and amplification were correlated with increased mRNA expression of *FOLR1* and all other genes (Supplementary Figure S6A). Higher mRNA expression was associated with DNA hypomethylation for *FOLR1* and all other genes except *SCL19A1*/RFC (Supplementary Figure S6B).

## 3. Discussion

In our study, we confirmed that *FOLR1* mRNA expression is correlated with protein expression in ovarian cancer cell lines and is high in cancer ovarian samples. Expression was heterogeneous between the cancer tissue samples with a relatively wide range of values (~6 units in log_2_), allowing the search for correlations with clinicopathological variables. We observed differences in *FOLR1* expression by histological type, tumor grade, and FIGO stage. Patients with high *FOLR1* expression were more likely to be diagnosed with serous histology, FIGO stage III–IV, and high-grade tumors; however, nearly similar percentages of patients with low *FOLR1* expression were also diagnosed with these features. These results are concordant with previously published studies [7,14,15]. To date, mirvetuximab soravtansine’s clinical development has largely been limited to high-grade serous platinum-resistant ovarian carcinomas. We show that high *FOLR1* mRNA expression was not differentially observed between platinum-sensitive and platinum-resistant tumors. It has been previously shown that FRα expression remains unchanged between diagnosis and relapse and between the primary tumor and metastatic foci, even after chemotherapy [4,10]. These data should encourage the further development of mirvetuximab soravtansine in the early and platinum sensitive setting, such as the PICCOLO trial (NCT05041257) and the ongoing GLORIOSA trial (NCT05445778). In addition, even though low-grade ovarian carcinomas are less associated with FRα overexpression, 21% in our cohort presented with high mRNA *FOLR1* expression. Using FRα immunohistochemistry on a tissue microarray, approximately 40% of ovarian low-grade serous carcinomas were FRα-high [16]. In this hard-to-treat histotype, future studies of FRα-directed therapy are warranted.

Several studies have described the expression of FRα in the serum of patients with ovarian carcinoma [17,18,19] and on circulating tumor cells [20]. FRα is known to shed from the cell surface into the circulation, which allows for its measurement in the serum of patients. FRα was significantly elevated in the serum of ovarian cancer patients compared to serum of both healthy controls and patients with benign gynecological conditions. Even though *FOLR1* was strongly correlated with CA125, it did not perform better than CA125 in early detection of ovarian cancer [18]. Serum FRα was associated with tumor FRα cell membrane expression and disease burden. Most importantly, high concentrations of serum FRα partly reduced anti-FRα antibody tumor cell killing [17]. Serum or circulating FRα seems to be a promising non-invasive tool for prognosis, and therapy-response monitoring as patients expressing higher levels may require more aggressive treatment. Integrating serum FRα and the ratio of serum/tumor FRα as a biomarker for future clinical trials assessing FRα-targeted therapies is essential.

A multivariate analysis of known prognostic factors in ovarian cancer (pathological type and grade, FIGO stage, macroscopic disease) revealed no association between *FOLR1* mRNA expression and survival at 6 months, 12 months, or more than 12 months. Previous studies evaluating the prognostic value of *FOLR1* expression in ovarian cancer have reported conflicting results [7,8,14,15], probably due to heterogeneity in methodology and the lack of inclusion of all known prognostic factors in the multivariate analyses. Our results are in agreement with Siu et al. [7] and Notaro et al. [14], showing no association between high FRα expression and overall or disease-free survival in high-grade serous/type 2 ovarian carcinoma in a multivariate analysis. In contrast, Köbel et al. [15] show an increase in overall survival among women with *FOLR1*-positive HGSC in the first two years of follow-up only, with a multivariate analysis not accounting for a major prognostic factor that is macroscopical residue.

We identified 187 robust genes whose expression was differential in tumors with *FOLR1* expression. Ontology analysis revealed that genes overexpressed in *FOLR1*-high samples were involved in transmembrane transport and the innate immune system. The glycoprotein FRα is a membrane-attached transporter that is highly expressed on a variety of cells, including cancer cells and activated macrophages. Once a folate conjugate is bound, it may be taken up via endocytosis, while a fraction remains engaged with the FR on the cell surface [21]. It has also been shown that anti-folate receptor-α IgE reprograms and recruits macrophages in the tumor microenvironment to attack tumors via TNF-α/CCL2 signaling [22]. In addition, FRα harbors immunogenic sequences, rendering it a suitable target for immunotherapeutic development in solid tumors. Various anti-FRα immunotherapeutic strategies, such as monoclonal antibodies, bispecific antibodies, CAR-T cells, and vaccines, are currently under development [23].

By contrast with *FOLR1* expression, the *FOLR1* high-like signature showed prognostic value, as it was significantly associated with improved survival independently from clinicopathological features in multivariate analysis. Integrating and validating the prognostic value of this signature in prospective trials is warranted.

High mRNA expression was associated with gene amplification and DNA hypomethylation for *FOLR1* and other genes included in the folate-signaling pathway, except *SCL19A1*/RFC. *FRα* gene amplification, as a possible mechanism of its overexpression, has been previously demonstrated [6,7]. This correlation could explain why FRα expression remains unchanged between diagnosis and relapse and between the primary tumor and metastatic foci, even after chemotherapy [4,10]. These findings suggest that *FOLR1* expression could be controlled genetically and epigenetically during ovarian cancer development and progression. However, Notaro et al. did not find a significant correlation between the expression of *FOLR1* and its specific promoter methylation. Their study focused on 254 ovarian cancers only and used MethyLight PCR reaction covering 7 of these 11 CpG sites upstream of the transcription site of *FOLR1* [14]. FRα and RFC, encoded by *SCL19A1,* have been described to have a paradoxical impact on cell proliferation, invasion, and clinical outcome in ovarian cancer. Reduced RFC expression was significantly associated with shorter overall survival and shorter disease-free survival, suggesting that RFC may be considered a marker for good prognosis in ovarian cancer patients. Among patients with high FRα-expressing ovarian cancers, overall survival and disease-free survival are significantly better in those with high RFC expression than in those without, indicating the protective role of RFC in patients with these tumors [7]. Whether RFC expression in high-FRα ovarian cancer patients treated with mirvetuximab soravtansine is prognostic needs to be evaluated in large prospective cohorts.

Our study displays several strengths: its originality, a large size of samples, and the biological and clinical relevance of *FOLR1* expression. It also includes a few limitations: its retrospective nature and associated biases, the mRNA assessment on bulk tissue samples rather than protein, and the lack of analysis of the correlation between mRNA and IHC protein expression in tumor tissues. Our analysis at the mRNA level allowed us to work on a very large series of samples annotated in terms of CLOVAR expression-based subtypes, and to search for associations with the expressions of other genes on a whole-genome scale. Even if we show good correlation between mRNA and protein expression levels for FRα in both pan-cancer and ovarian cancer cell lines, further validation at the protein level directly on clinical samples remains warranted.

In conclusion, we identified a prognostic *FOLR1* high-like signature based on 187 genes that was significantly associated with improved survival, independently from clinicopathological features. Evaluating the prognostic and predictive value of the *FOLR1* high-like signature and of RFC expression in large prospective cohorts of ovarian cancer patients treated with mirvetuximab soravtansine and novel strategies is warranted.

## 4. Material and Methods

### 4.1. Ovarian Cancer Samples and Gene Expression Profiling

We analyzed our ovarian cancer gene expression data [24] pooled with 15 public transcriptome datasets of OC with associated clinicopathological annotations [25,26,27,28,29,30,31,32,33,34,35,36,37,38]. The collected database comprised 1832 non-redundant and primary OC samples, including 38 from our institution and 30 normal ovarian tissue samples. These datasets were collected from the National Center for Biotechnology Information (NCBI)/GenBank GEO and The Cancer Genome Atlas portal (TCGA) databases (Supplementary Table S1). The samples were profiled using DNA microarrays (Affymetrix, Agilent, and spotted cDNA microarrays, Santa Clara, CA, USA) and RNA sequencing (Illumina, Illumina Inc., San Diego, CA, USA). The details of the Institutional Review Board and Ethical Committee approval and patient consent for all 16 studies are presented in their corresponding publications listed in Supplementary Table S1.

### 4.2. Gene and Protein Expression Profile on Cancer Cell Lines

We also collected and analyzed the gene expression and protein expression data of 363 cancer cell lines, including 17 ovarian cell lines, from the Broad Institute Cancer Cell Line Encyclopedia (CCLE) [39]. This dataset is publicly available through the Broad Institute and is hosted on the Cancer Dependency Map portal (DepMap, 21Q4 version). Gene expression data were obtained using RNA sequencing (RNA-seq) and normalized using the Transcripts Per Million (TPM) method. Protein expression data were acquired using Reverse Phase Protein Arrays (RPPA), a high-throughput, antibody-based technique that quantifies the relative expression levels of proteins. Pearson’s correlation test was applied to assess the correlation between gene and protein expression.

### 4.3. Gene Expression Data Analysis

Before analysis, several steps of data processing were applied. The first step was the normalization of each dataset separately. It was conducted using the R software (version 4.2.1) using Bioconductor and associated packages; we used quantile normalization for the already processed data from non-Affymetrix-based sets (Agilent, SweGene, and Operon) and Robust Multichip Average (RMA) with the non-parametric quantile algorithm for the raw Affymetrix data. In the second step, we mapped the hybridization probes across the different DNA microarray platforms represented as previously reported [40]. When multiple probes mapped to the same Entrez Gene ID, we retained the most variant probe in a particular dataset. We log_2_-transformed the available RNA-Seq data that were already normalized. Before analysis, gene expression levels were standardized within each dataset, and the primary OC population was used as a reference. This allowed the exclusion of biases due to laboratory-specific variations and population heterogeneity, and to make data comparable across all sets. *FOLR1* expression in tumors was measured as a discrete value (high vs. low) by using the median expression level across the whole series as the cut-off. The same discretization was applied for each gene analyzed individually. The CLOVAR (Classification of Ovarian Cancer) molecular subtypes of OC were defined as described [13]. We also collected DNA copy number (array-CGH) and CpG DNA methylation data of *FOLR1* from TCGA [33] to assess their correlation with *FORL1* mRNA expression. To decipher the biological pathways associated with *FOLR1* expression in OC, we applied a supervised analysis to the expression profiles of the 571 TCGA OC samples (learning set) to search for genes differentially expressed between the “*FOLR1*-high” and “*FOLR1*-low” expression classes. We used a moderated t test with empirical Bayes statistics included in the limma R packages. False discovery rate (Hochberg and Benjamini, 1990) was applied to correct the multiple testing hypothesis: The significant genes were defined by *p* < 5%, q < 1%, and a fold change (FC) greater than |1.25x|. To ensure the robustness of the identified gene list, we validated it using an independent set of the remaining 1261 OC samples (631 “*FOLR1*-low” and 630 “*FOLR1*-high” samples). We computed a “*FOLR1*-high” median profile based on the significant genes from the standardized learning set. Pearson correlation distances were then measured between this “*FOLR1*-high” profile and each sample in each standardized validation set. Samples were then classified as “*FOLR1*-high like” or “*FOLR1*-low like” on the basis of positive or negative Pearson correlation coefficients, respectively. Ontology analysis of the resulting gene list was based on the REACTOME Database for Annotation, Visualization and Integrated Discovery (DAVID; “http://david.abcc.ncifcrf.gov/ (accessed on 12 January 2024)”.

### 4.4. Statistical Analysis

Correlations between the tumor groups and clinicopathological features were analyzed using the t-test or the Fisher’s exact test (variables with two groups) when appropriate or one-way analysis of variance (ANOVA; variables with more than two groups). Progression-free survival (PFS) was calculated from the date of diagnosis until the date of relapse or death, whichever occurred first. Overall survival (OS) was calculated from the date of diagnosis to the date of death. Follow-up was measured from the date of diagnosis to the date of last news for event-free patients. Survival was calculated using the Kaplan–Meier method, and curves were compared using the log-rank test. Univariate and multivariate survival analyses were performed using Cox regression analysis (Wald test). Variables tested in univariate analyses included pathological type, stage, grade, and presence of macroscopic residual disease after surgery. Variables with a *p*-value < 0.05 in the univariate analysis were tested in the multivariate analysis. Differences in the prognostic effect of *FOLR1* expression by molecular subtype were assessed using a Cox model with an interaction term between expression and subtype. All the statistical tests were two-sided at the 5% level of significance. Statistical analysis was performed using the R software (version 4.2.1).

## Figures and Tables

**Table 1:** *FOLR1* mRNA expression and clinicopathological features.

Characteristic	N	All, N (%)	*FOLR1* mRNA Classes		*p* Value	Odds-Ratio
			Low	High		
Age, year median (range)	1398	59 (21–90)	59.6 (21–88)	59.7 (22–89.95)	0.832	
NA	434					
Pathological type					3.80 × 10^−4^	2.45
Serous	929	929 (92%)	449 (89%)	480 (95%)		[1.46–4.21]
Clear cell	20	20 (2%)	15 (3%)	5 (1%)		
Endometrioid	29	29 (3%)	18 (4%)	11 (2%)		
Mucinous	18	18 (2%)	17 (3%)	1 (0%)		
Other	12	12 (1%)	5 (1%)	7 (1%)		
NA	824					
FIGO stage					2.16 × 10^−3^	1.71
I–II	162	162 (12%)	100 (15%)	62 (9%)		[1.21–2.44]
III–IV	1195	1195 (88%)	580 (85%)	615 (91%)		
NA	475					
Pathological grade					2.24 × 10^−4^	1.45
Low	343	343 (25%)	194 (28%)	149 (21%)		[1.12–1.87]
High	1041	1041 (75%)	493 (71%)	548 (79%)		
NA	448					
Macroscopic disease after surgery					0.440	1.1
No	446	446 (33%)	230 (34%)	216 (32%)		[0.87–1.39]
Yes	899	899 (67%)	442 (66%)	457 (68%)		
NA	487					
Response to platinum					0.916	1.03
Resistant	158	158 (12%)	79 (12%)	79 (12%)		[0.73–1.46]
Sensitive	1183	1183 (88%)	582 (88%)	601 (88%)		
NA	491					
*TP53* status					0.967	1.17
Wild-type	15	15 (3%)	8 (3%)	7 (3%)		[0.36–3.87]
Mutated	456	456 (97%)	225 (97%)	231 (97%)		
NA	1361					
*BRCA1/2* status					0.107	1.48
Wild-type	374	374 (78%)	190 (81%)	184 (75%)		[0.93–2.35]
Mutated	105	105 (22%)	44 (19%)	61 (25%)		
NA	1353					
CLOVAR subtype					2.70 × 10^−14^	
Differentiated	521	521 (28%)	220 (24%)	301 (33%)		
Immunoreactive	419	419 (23%)	163 (18%)	256 (28%)		
Mesenchymal	435	435 (24%)	268 (29%)	167 (18%)		
Proliferative	454	454 (25%)	260 (29%)	194 (21%)		
NA	3					
Median follow-up, months (range)	1471	35.8 (1–243)	33 (1–243)	36.7 (1–230)	0.916	
Median OS, month (range)	1471	45 (1–243)	43 (1–243)	47 (1–230)	0.953	
Median PFS, month (range)	1207	35 (1–243)	34 (1–243)	36 (1–230)	0.696	

*FOLR1* expression was significantly higher in OC samples than in normal ovarian samples (OR = 3.88 [2.64–5.70], *p* = 6.97 × 10^−12^) (Figure 1A).

## Data Availability

All the data generated or analyzed during this study are included in this published article and its Supplementary Information files.

## Supplementary Materials

The following supporting information can be downloaded at https://www.mdpi.com/article/10.3390/ijms252211953/s1.

## Abbreviations

DHFR	Dihydrofolate reductase
FOLH1	Glutamate carboxypeptidase
FR	Folate receptor
FRα	Folate receptor alpha
mRNA	Messenger RNA
MTHFR	Methylene tetrahydrofolate reductase
OS	Overall survival
PFS	Progression-free survival
RFC	Reduced folate carrier
SLC19A1	Folate transporter 1
TYMS	Thymidylate synthase
