# Supplementary material for: Deciphering Folate Receptor alphaGene Expression and mRNA Signatures in Ovarian Cancer: Implications for Precision Therapies
Source: Int J Mol Sci. 2024 Nov 7;25(22):11953. doi: 10.3390/ijms252211953 (PMC11593678; doi:10.3390/ijms252211953)
Supplement: Supplementary file 1 [file ijms-25-11953-s001.zip › ijms-3205810-supplementary.pdf]

Supplementary material

Supplementary Table S1: List of ovarian cancer data sets included in our analysis

Reference	Source of data	Technological platform	N° of probe sets/genes	All samples	Normal	Primary
[Partheen K et al., Eur. J. Cancer 2006]	GEO database, GSE12418	Swegene, H_v2.1.1_27K	15K	54	0	54
[Moreno C et al., PloS one 2007]	GEO database, GSE7463	Affymetrix, array U95Av2	13K	43	0	33
[Bonome T et al., Cancer Res 2008]	GEO database, GSE26712	Affymetrix, array U133 A	22K	195	10	185
[Tothill R et al., Clin Cancer Res 2008]	GEO database, GSE9899	Affymetrix, array U133 Plus 2.0	54K	295	0	285
[Crijns A et al., PLoS Med 2009]	GEO database, GSE13876	Central Microarray Facility, Operon human v3	15K	157	0	157
[Denkert C et al., J Pathol 2009]	GEO database, GSE14767	Affymetrix, array U133 A	22K	80	0	79
[Marchion D et al., Clin Cancer Res 2011]	GEO database, GSE23554	Affymetrix, array U133 A	22K	28	0	28
[Mateescu B et al., Nat. Med. 2011]	GEO database, GSE26193	Affymetrix, array U133 Plus 2.0	54K	107	0	104
[Sabatier et al., Br. J. Cancer 2011]	Unregistered	Affymetrix, Human Exon 1.0 ST	22K	55	2	38
[TCGA, Nature 2011]	TCGA Data Portal – OVCA –	Affymetrix, Human Exon 1.0 ST	22K	600	10	571
[Koti M et al., BMC Cancer 2013]	GEO database, GSE51373	Affymetrix, array U133 Plus 2.0	54K	28	0	28
[Karlan BY et al., Gynecol. Oncol. 2014]	GEO database, GSE51088	Agilent, array Human 1A (012097)	20K	172	0	104
[Lisowska K et al., Front. Oncol. 2014]	GEO database, GSE63885	Affymetrix, array U133 Plus 2.0	54K	101	0	101
[Mitra S et al, Cancers 2019]	GEO database, GSE137237/137238	Illumina, RNA sequencing	58K	38	8	19
[Artibani M et al., JCI Insight. 2021]	GEO database, GSE162714	Illumina, RNA sequencing	58K	77	0	16
[Lee JY et al., GEO (2023)]	GEO database, GSE230541	Illumina, RNA sequencing	22K	30	0	30
**Total**					30	1832

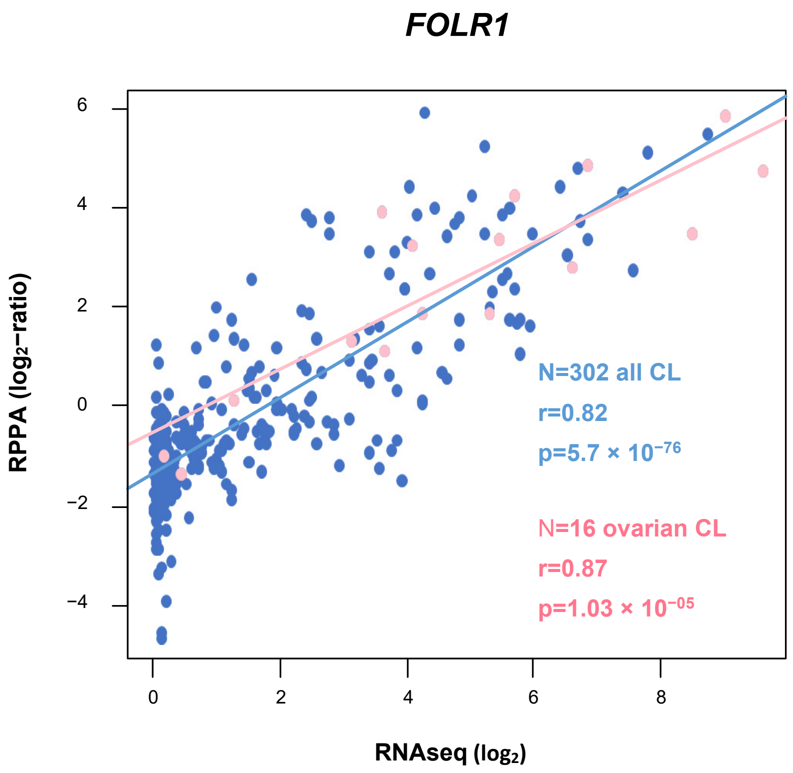

Supplementary Figure S1: Scatterplot of FOLR1 mRNA and protein expression levels in CCLE cell lines.

Pearson correlation test was used to assess the correlation in all cell lines (blue) and ovarian cell lines (pink).

Abbreviations: CL: cell lines, CCLE: Cancer Cell Line Encyclopedia

A

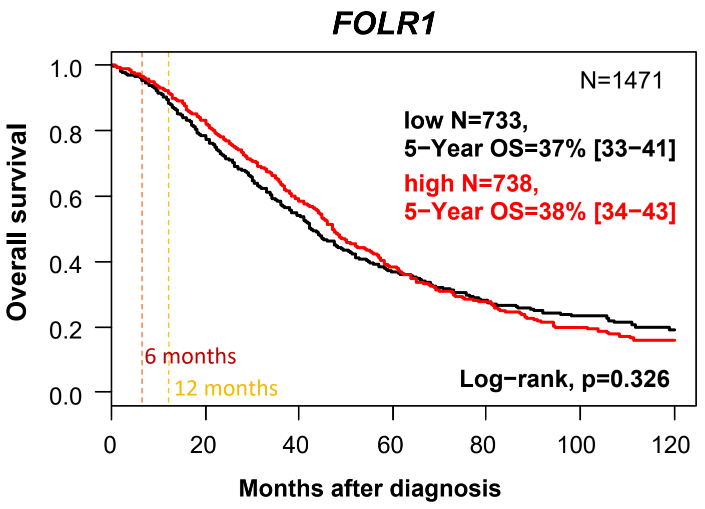

B

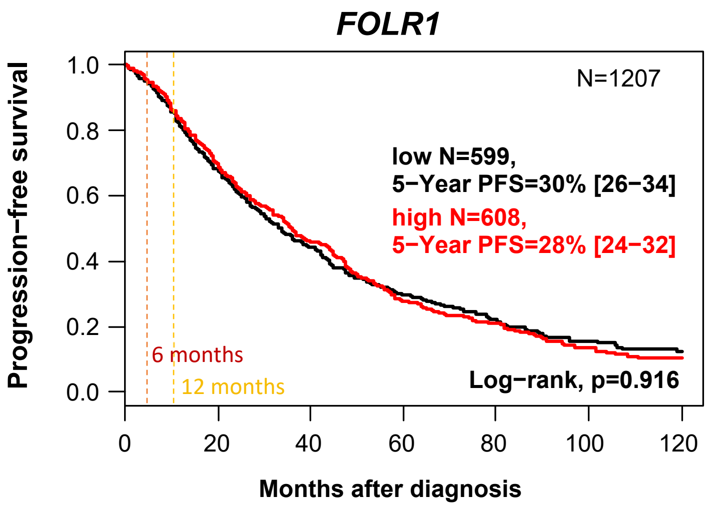

Supplementary Figure S2: Correlation of *FOLR1* mRNA expression with survival in a time−dependent analysis at 6, 12 and beyond 12−monts.

(A) Kaplan Meier curve for progression−free survival according to FOLR1 mRNA expression in all patients (B) Kaplan Meier curve for overall survival according to FOLR1 mRNA expression in all patients.

A

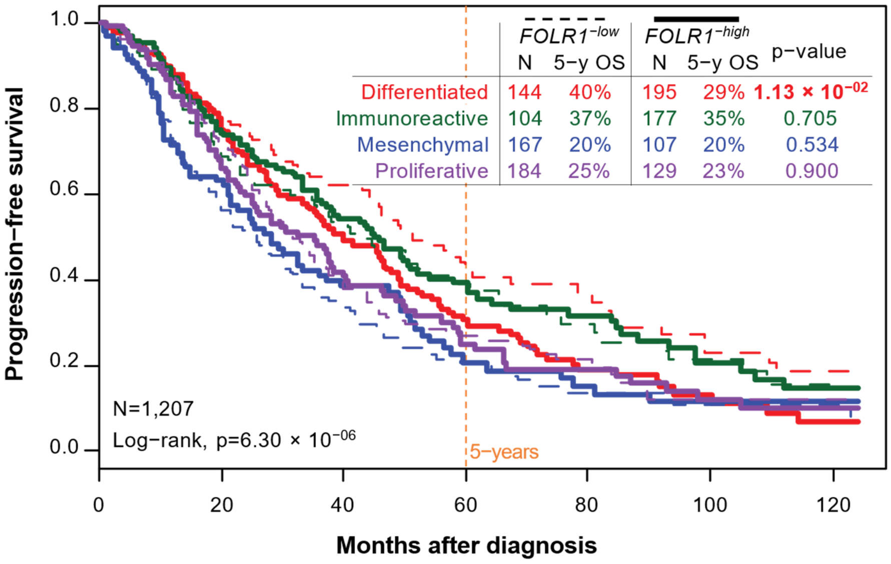

B

PFS, differentiated* OC	Univariate N	Univariate HR [95%CI]	Univariate p−value	Multivariate N	Multivariate HR [95%CI]	Multivariate p−value
Pathological type, serous vs. non−serous	93	2.89 [1.37−6.11]	5.48×10^{-03}	54	0.81 [0.28−2.30]	0.689
FIGO stage, III−IV vs. I−II	274	5.12 [3.06−8.59]	5.65×10^{-10}	54	9.49 [3.07−29.3]	9.40×10^{-05}
Pathological grade, high vs. low	267	1.58 [1.10−2.25]	1.26×10^{-02}	54	1.91 [0.92−3.97]	0.081
Macroscopic disease, yes vs. no	281	2.14 [1.55−2.96]	4.25×10^{-06}	54	1.03 [0.42−2.48]	0.953
FOLR1 classes, high vs. low	339	1.44 [1.08−1.91]	1.18×10^{-02}	54	1.20 [0.61−2.37]	0.595

** CLOVAR subtype*

Supplementary Figure S3: Correlation of *FOLR1* mRNA expression with survival *per* CLOVAR molecular subtype

(A) Kaplan Meier curve for progression−free survival according to FOLR1 mRNA expression in CLOVAR molecular subtypes, significance assessed by using Log−rank. (B) Uni− and Multivariate Cox regression analysis of progression−free survival according to FOLR1 mRNA expression in the differentiated CLOVAR subtype and clinicopathological features, significance assessed by using Wald’s test.

Supplementary Table S2: List of 187 genes differentially expressed between ovarian cancers with versus without FOLR1 high expression.

Gene Symbol	Gene Title	Chromosomal Location	Gene ID	Ratio fold (log2)	t	*P*-value	*Q*-value
FOLR1	folate receptor 1 (adult)	11q13.3–q14.1	2348	1,61	27,63	$1{,}51 \times 10^{-107}$	$9{,}51 \times 10^{-104}$
FOLR3	folate receptor 3 (gamma)	11q13	2352	0,71	11,45	$1{,}69 \times 10^{-27}$	$5{,}32 \times 10^{-24}$
MSLN	mesothelin	16p13.3	10232	0,63	8,80	$1{,}63 \times 10^{-17}$	$3{,}43 \times 10^{-14}$
GSTK1	glutathione S−transferase kappa 1	7q35	373156	0,35	7,83	$2{,}31 \times 10^{-14}$	$1{,}82 \times 10^{-11}$
S100A1	S100 calcium binding protein A1	1q21	6271	0,66	7,44	$3{,}72 \times 10^{-13}$	$1{,}70 \times 10^{-10}$
CD82	CD82 molecule	11p11.2	3732	0,37	7,39	$5{,}14 \times 10^{-13}$	$2{,}05 \times 10^{-10}$
SNCG	synuclein, gamma (breast cancer−specific protein 1)	10q23.2−q23.3	6623	0,44	7,27	$1{,}17 \times 10^{-12}$	$3{,}90 \times 10^{-10}$
RARRES3	retinoic acid receptor responder (tazarotene induced) 3	11q23	5920	0,64	7,07	$4{,}40 \times 10^{-12}$	$1{,}12 \times 10^{-09}$
CRYAB	crystallin, alpha B	11q22.3−q23.1	1410	0,77	7,06	$4{,}97 \times 10^{-12}$	$1{,}12 \times 10^{-09}$
MUC20	mucin 20, cell surface associated	3q29	200958	0,44	7,00	$7{,}03 \times 10^{-12}$	$1{,}42 \times 10^{-09}$
CLU	clusterin	8p21−p12	1191	0,51	6,92	$1{,}21 \times 10^{-11}$	$2{,}12 \times 10^{-09}$
C10orf116	chromosome 10 open reading frame 116	10q23.2	10974	0,42	6,88	$1{,}62 \times 10^{-11}$	$2{,}61 \times 10^{-09}$
EPHX1	epoxide hydrolase 1, microsomal (xenobiotic)	1q42.1	2052	0,34	6,66	$6{,}58 \times 10^{-11}$	$8{,}67 \times 10^{-09}$
S100A14	S100 calcium binding protein A14	1q21.3	57402	0,56	6,62	$8{,}16 \times 10^{-11}$	$1{,}01 \times 10^{-08}$
SCGB1D2	secretoglobin, family 1D, member 2	11q13	10647	0,82	6,60	$9{,}65 \times 10^{-11}$	$1{,}11 \times 10^{-08}$
SLC6A12	solute carrier family 6 (neurotransmitter transporter, betaine/GABA), member 12	12p13	6539	0,40	6,57	$1{,}16 \times 10^{-10}$	$1{,}31 \times 10^{-08}$
SLC4A11	solute carrier family 4, sodium borate transporter, member 11	20p12	83959	0,44	6,50	$1{,}79 \times 10^{-10}$	$1{,}98 \times 10^{-08}$
UCP2	uncoupling protein 2 (mitochondrial, proton carrier)	11q13	7351	0,40	6,48	$1{,}98 \times 10^{-10}$	$2{,}09 \times 10^{-08}$
CYP4B1	cytochrome P450, family 4, subfamily B, polypeptide 1	1p34−p12	1580	0,70	6,44	$2{,}58 \times 10^{-10}$	$2{,}58 \times 10^{-08}$
ALKBH7	alkB, alkylation repair homolog 7 (E. coli)	19p13.3	84266	0,32	6,38	$3{,}75 \times 10^{-10}$	$3{,}48 \times 10^{-08}$
C21orf63	chromosome 21 open reading frame 63	21q22.11	59271	0,54	6,37	$3{,}82 \times 10^{-10}$	$3{,}50 \times 10^{-08}$
PDZK1IP1	PDZK1 interacting protein 1	1p33	10158	0,60	6,33	$4{,}95 \times 10^{-10}$	$4{,}46 \times 10^{-08}$
HSPB2	heat shock 27kDa protein 2	11q22−q23	3316	0,37	6,33	$5{,}02 \times 10^{-10}$	$4{,}46 \times 10^{-08}$
NDUFB7	NADH dehydrogenase (ubiquinone) 1 beta subcomplex, 7, 18kDa	19p13.12−p13.11	4713	0,33	6,32	$5{,}36 \times 10^{-10}$	$4{,}70 \times 10^{-08}$
S100A13	S100 calcium binding protein A13	1q21	6284	0,44	6,31	$5{,}51 \times 10^{-10}$	$4{,}74 \times 10^{-08}$

SELENBP1	selenium binding protein 1	1q21−q22	8991	0,37	6,15	$1{,}42 \times 10^{-09}$	$9{,}95 \times 10^{-08}$
GJB1	gap junction protein, beta 1, 32kDa	Xq13.1	2705	0,43	6,13	$1{,}64 \times 10^{-09}$	$1{,}14 \times 10^{-07}$
DEGS2	delta 4−desaturase, sphingolipid 2	14q32.2	123099	0,33	6,07	$2{,}28 \times 10^{-09}$	$1{,}43 \times 10^{-07}$
UPK3B	uroplakin 3B	7q11.2	80761	0,40	6,03	$3{,}01 \times 10^{-09}$	$1{,}83 \times 10^{-07}$
KRT7	keratin 7	12q12−q13	3855	0,37	6,01	$3{,}28 \times 10^{-09}$	$1{,}93 \times 10^{-07}$
SCARA3	scavenger receptor class A, member 3	8p21	51435	0,33	5,97	$4{,}23 \times 10^{-09}$	$2{,}30 \times 10^{-07}$
ATP6V1B1	ATPase, H+ transporting, lysosomal 56/58kDa, V1 subunit B1	2p13.1	525	0,51	5,92	$5{,}61 \times 10^{-09}$	$2{,}90 \times 10^{-07}$
DEFB1	defensin, beta 1	8p23.2−p23.1	1672	0,89	5,91	$5{,}75 \times 10^{-09}$	$2{,}95 \times 10^{-07}$
DHCR24	24−dehydrocholesterol reductase	1p33−p31.1	1718	0,38	5,86	$7{,}82 \times 10^{-09}$	$3{,}63 \times 10^{-07}$
CLDN3	claudin 3	7q11.23	1365	0,34	5,86	$7{,}83 \times 10^{-09}$	$3{,}63 \times 10^{-07}$
KLK8	kallikrein−related peptidase 8	19q13.3−q13.4	11202	0,38	5,81	$1{,}05 \times 10^{-08}$	$4{,}53 \times 10^{-07}$
MAL	mal, T−cell differentiation protein	2cen−q13	4118	0,57	5,76	$1{,}37 \times 10^{-08}$	$5{,}75 \times 10^{-07}$
S100A6	S100 calcium binding protein A6	1q21	6277	0,35	5,74	$1{,}56 \times 10^{-08}$	$6{,}34 \times 10^{-07}$
RSPO1	R−spondin homolog (Xenopus laevis)	1p34.3	284654	0,44	5,72	$1{,}73 \times 10^{-08}$	$6{,}84 \times 10^{-07}$
GAB2	GRB2−associated binding protein 2	11q14.1	9846	0,39	5,71	$1{,}77 \times 10^{-08}$	$6{,}91 \times 10^{-07}$
GAS6	growth arrest−specific 6	13q34	2621	0,34	5,66	$2{,}42 \times 10^{-08}$	$8{,}82 \times 10^{-07}$
TMPRSS3	transmembrane protease, serine 3	21q22.3	64699	0,46	5,63	$2{,}75 \times 10^{-08}$	$9{,}92 \times 10^{-07}$
S100A4	S100 calcium binding protein A4	1q21	6275	0,50	5,61	$3{,}08 \times 10^{-08}$	$1{,}09 \times 10^{-06}$
LCN2	lipocalin 2	9q34	3934	0,67	5,61	$3{,}18 \times 10^{-08}$	$1{,}10 \times 10^{-06}$
DOK5	docking protein 5	20q13.2	55816	0,60	5,59	$3{,}43 \times 10^{-08}$	$1{,}17 \times 10^{-06}$
CHI3L1	chitinase 3−like 1 (cartilage glycoprotein−39)	1q32.1	1116	0,50	5,55	$4{,}38 \times 10^{-08}$	$1{,}42 \times 10^{-06}$
C11orf67	chromosome 11 open reading frame 67	11q14.1	28971	0,32	5,50	$5{,}86 \times 10^{-08}$	$1{,}84 \times 10^{-06}$
BBOX1	butyrobetaine (gamma), 2−oxoglutarate dioxygenase (gamma−butyrobetaine hydroxylase) 1	11p14.2	8424	0,63	5,48	$6{,}49 \times 10^{-08}$	$1{,}97 \times 10^{-06}$
UGT2B7	UDP glucuronosyltransferase 2 family, polypeptide B7	4q13	7364	0,46	5,45	$7{,}47 \times 10^{-08}$	$2{,}20 \times 10^{-06}$
SKAP1	src kinase associated phosphoprotein 1	17q21.32	8631	0,34	5,38	$1{,}07 \times 10^{-07}$	$2{,}89 \times 10^{-06}$
CLYBL	citrate lyase beta like	13q32	171425	0,35	5,33	$1{,}39 \times 10^{-07}$	$3{,}59 \times 10^{-06}$
SLPI	secretory leukocyte peptidase inhibitor	20q12	6590	0,45	5,31	$1{,}53 \times 10^{-07}$	$3{,}80 \times 10^{-06}$
NPR1	natriuretic peptide receptor A/guanylate cyclase A (atrionatriuretic peptide receptor A)	1q21−q22	4881	0,38	5,28	$1{,}79 \times 10^{-07}$	$4{,}26 \times 10^{-06}$
SCGB2A1	secretoglobin, family 2A, member 1	11q13	4246	0,50	5,25	$2{,}14 \times 10^{-07}$	$4{,}79 \times 10^{-06}$
ZBED2	zinc finger, BED−type containing 2	3q13.13	79413	0,47	5,25	$2{,}19 \times 10^{-07}$	$4{,}85 \times 10^{-06}$
CFB	complement factor B	6p21.3	629	0,47	5,23	$2{,}42 \times 10^{-07}$	$5{,}21 \times 10^{-06}$

PSMB10	proteasome (prosome, macropain) subunit, beta type, 10	16q22.1	5699	0,33	5,14	$3{,}84 \times 10^{-07}$	$7{,}57 \times 10^{-06}$
TMEM211	Transmembrane protein 211	22q11.23	255349	0,35	5,06	$5{,}59 \times 10^{-07}$	$1{,}02 \times 10^{-05}$
CXCL17	C−X−C motif chemokine ligand 17	19q13.2	284340	0,58	5,05	$5{,}90 \times 10^{-07}$	$1{,}06 \times 10^{-05}$
HLA−DOB	major histocompatibility complex, class II, DO beta	6p21.3	3112	0,33	5,00	$7{,}53 \times 10^{-07}$	$1{,}28 \times 10^{-05}$
APOA1	apolipoprotein A−I	11q23−q24	335	0,49	4,99	$7{,}97 \times 10^{-07}$	$1{,}34 \times 10^{-05}$
KLK7	kallikrein−related peptidase 7	19q13.33	5650	0,42	4,98	$8{,}25 \times 10^{-07}$	$1{,}36 \times 10^{-05}$
ERBB4	v−erb−a erythroblastic leukemia viral oncogene homolog 4 (avian)	2q33.3−q34	2066	0,52	4,98	$8{,}32 \times 10^{-07}$	$1{,}36 \times 10^{-05}$
PNOC	prepronociceptin	8p21	5368	0,47	4,96	$9{,}27 \times 10^{-07}$	$1{,}48 \times 10^{-05}$
PI3	peptidase inhibitor 3, skin−derived	20q12−q13	5266	0,61	4,94	$1{,}02 \times 10^{-06}$	$1{,}59 \times 10^{-05}$
SLC15A2	solute carrier family 15 (H+/peptide transporter), member 2	3q13.33	6565	0,50	4,87	$1{,}44 \times 10^{-06}$	$2{,}06 \times 10^{-05}$
SLC1A3	solute carrier family 1 (glial high affinity glutamate transporter), member 3	5p13	6507	0,34	4,84	$1{,}67 \times 10^{-06}$	$2{,}33 \times 10^{-05}$
HTR3A	5−hydroxytryptamine (serotonin) receptor 3A	11q23.1	3359	0,49	4,82	$1{,}80 \times 10^{-06}$	$2{,}47 \times 10^{-05}$
PLA2G16	phospholipase A and acyltransferase 3	11q12.3−q13.1	11145	0,35	4,82	$1{,}83 \times 10^{-06}$	$2{,}49 \times 10^{-05}$
GPR110	G protein−coupled receptor 110	6p12.3	266977	0,42	4,75	$2{,}59 \times 10^{-06}$	$3{,}28 \times 10^{-05}$
TSPAN12	tetraspanin 12	7q31.31	23554	0,40	4,75	$2{,}61 \times 10^{-06}$	$3{,}29 \times 10^{-05}$
LAMA3	laminin, alpha 3	18q11.2	3909	0,33	4,73	$2{,}81 \times 10^{-06}$	$3{,}47 \times 10^{-05}$
DEFB4	defensin, beta 4	8p23.1−p22	1673	0,33	4,65	$4{,}07 \times 10^{-06}$	$4{,}62 \times 10^{-05}$
MUC1	mucin 1, cell surface associated	1q21	4582	0,36	4,63	$4{,}51 \times 10^{-06}$	$4{,}99 \times 10^{-05}$
TGM1	transglutaminase 1 (K polypeptide epidermal type I, protein−glutamine−gamma−glutamyltransferase)	14q11.2	7051	0,42	4,62	$4{,}66 \times 10^{-06}$	$5{,}12 \times 10^{-05}$
KLK10	kallikrein−related peptidase 10	19q13.3−q13.4	5655	0,34	4,62	$4{,}76 \times 10^{-06}$	$5{,}20 \times 10^{-05}$
EHF	ets homologous factor	11p12	26298	0,37	4,61	$5{,}07 \times 10^{-06}$	$5{,}43 \times 10^{-05}$
VGLL1	vestigial like 1 (Drosophila)	Xq26.3	51442	0,45	4,59	$5{,}35 \times 10^{-06}$	$5{,}64 \times 10^{-05}$
ACSL5	acyl−CoA synthetase long−chain family member 5	10q25.1−q25.2	51703	0,37	4,58	$5{,}67 \times 10^{-06}$	$5{,}90 \times 10^{-05}$
MEOX1	mesenchyme homeobox 1	17q21	4222	0,34	4,58	$5{,}82 \times 10^{-06}$	$6{,}01 \times 10^{-05}$
LY6G6C	lymphocyte antigen 6 complex, locus G6C	6p21.33	80740	0,39	4,56	$6{,}17 \times 10^{-06}$	$6{,}23 \times 10^{-05}$
CYP4F11	cytochrome P450, family 4, subfamily F, polypeptide 11	19p13.1	57834	0,34	4,54	$6{,}89 \times 10^{-06}$	$6{,}85 \times 10^{-05}$
CD74	CD74 molecule, major histocompatibility complex, class II invariant chain	5q32	972	0,37	4,51	$7{,}91 \times 10^{-06}$	$7{,}61 \times 10^{-05}$
TACSTD2	tumor−associated calcium signal transducer 2	1p32−p31	4070	0,35	4,50	$8{,}39 \times 10^{-06}$	$7{,}94 \times 10^{-05}$

EYA2	eyes absent homolog 2 (Drosophila)	20q13.1	2139	0,42	4,46	$9{,}81 \times 10^{-06}$	$9{,}01 \times 10^{-05}$
GMPR	guanosine monophosphate reductase	6p23	2766	0,35	4,46	$9{,}90 \times 10^{-06}$	$9{,}07 \times 10^{-05}$
CHODL	chondrolectin	21q11.2	140578	0,35	4,45	$1{,}03 \times 10^{-05}$	$9{,}23 \times 10^{-05}$
TSPAN1	tetraspanin 1	1p34.1	10103	0,59	4,38	$1{,}40 \times 10^{-05}$	$1{,}15 \times 10^{-04}$
HGD	homogentisate 1,2−dioxygenase (homogentisate oxidase)	3q13.33	3081	0,42	4,32	$1{,}83 \times 10^{-05}$	$1{,}42 \times 10^{-04}$
CP	ceruloplasmin (ferroxidase)	3q23−q25	1356	0,48	4,29	$2{,}09 \times 10^{-05}$	$1{,}55 \times 10^{-04}$
OVGP1	oviductal glycoprotein 1, 120kDa	1p13	5016	0,58	4,17	$3{,}50 \times 10^{-05}$	$2{,}28 \times 10^{-04}$
RERG	RAS−like, estrogen−regulated, growth inhibitor	12p12.3	85004	0,42	4,17	$3{,}58 \times 10^{-05}$	$2{,}32 \times 10^{-04}$
SCGB1D1	secretoglobin, family 1D, member 1	11q13	10648	0,48	4,13	$4{,}20 \times 10^{-05}$	$2{,}62 \times 10^{-04}$
FAM3B	family with sequence similarity 3, member B	21q22.3	54097	0,37	4,09	$4{,}88 \times 10^{-05}$	$2{,}93 \times 10^{-04}$
HLA−DMA	major histocompatibility complex, class II, DM alpha	6p21.3	3108	0,39	4,07	$5{,}43 \times 10^{-05}$	$3{,}20 \times 10^{-04}$
GLDC	glycine dehydrogenase (decarboxylating)	9p22	2731	0,42	4,04	$6{,}02 \times 10^{-05}$	$3{,}44 \times 10^{-04}$
HLA−DMB	major histocompatibility complex, class II, DM beta	6p21.3	3109	0,34	3,96	$8{,}34 \times 10^{-05}$	$4{,}35 \times 10^{-04}$
PIGR	polymeric immunoglobulin receptor	1q31−q41	5284	0,43	3,90	$1{,}06 \times 10^{-04}$	$5{,}19 \times 10^{-04}$
CRIP1	cysteine−rich protein 1 (intestinal)	14q32.33	1396	0,37	3,90	$1{,}09 \times 10^{-04}$	$5{,}28 \times 10^{-04}$
FABP3	fatty acid binding protein 3, muscle and heart (mammary−derived growth inhibitor)	1p33−p32	2170	0,35	3,80	$1{,}58 \times 10^{-04}$	$6{,}91 \times 10^{-04}$
UCA1	urothelial cancer associated 1	19p13.12	652995	0,45	3,75	$1{,}96 \times 10^{-04}$	$8{,}13 \times 10^{-04}$
KLK5	kallikrein−related peptidase 5	19q13.3−q13.4	25818	0,38	3,74	$2{,}03 \times 10^{-04}$	$8{,}32 \times 10^{-04}$
SCGB1A1	secretoglobin, family 1A, member 1 (uteroglobin)	11q12.3−q13.1	7356	0,41	3,72	$2{,}20 \times 10^{-04}$	$8{,}81 \times 10^{-04}$
CAPS	calcyphosine	19p13.3	828	0,33	3,71	$2{,}30 \times 10^{-04}$	$9{,}08 \times 10^{-04}$
SLC34A2	solute carrier family 34 (sodium phosphate), member 2	4p15.3−p15.1	10568	0,37	3,71	$2{,}31 \times 10^{-04}$	$9{,}10 \times 10^{-04}$
HLA−DRA	major histocompatibility complex, class II, DR alpha	6p21.3	3122	0,33	3,70	$2{,}38 \times 10^{-04}$	$9{,}29 \times 10^{-04}$
BST2	bone marrow stromal cell antigen 2	19p13.2	684	0,36	3,62	$3{,}15 \times 10^{-04}$	$1{,}14 \times 10^{-03}$
MUC15	mucin 15, cell surface associated	11p14.3	143662	0,43	3,58	$3{,}72 \times 10^{-04}$	$1{,}28 \times 10^{-03}$
LTF	lactotransferrin	3p21.31	4057	0,46	3,57	$3{,}85 \times 10^{-04}$	$1{,}31 \times 10^{-03}$
MMP7	matrix metallopeptidase 7 (matrilysin, uterine)	11q21−q22	4316	0,55	3,53	$4{,}46 \times 10^{-04}$	$1{,}45 \times 10^{-03}$
HPGD	hydroxyprostaglandin dehydrogenase 15−(NAD)	4q34−q35	3248	0,45	3,47	$5{,}58 \times 10^{-04}$	$1{,}70 \times 10^{-03}$
CRTAC1	cartilage acidic protein 1	10q22	55118	0,33	3,45	$5{,}95 \times 10^{-04}$	$1{,}79 \times 10^{-03}$
SCGB2A2	secretoglobin, family 2A, member 2	11q13	4250	0,40	3,43	$6{,}36 \times 10^{-04}$	$1{,}87 \times 10^{-03}$
AADAC	arylacetamide deacetylase (esterase)	3q21.3−q25.2	13	0,33	3,42	$6{,}73 \times 10^{-04}$	$1{,}94 \times 10^{-03}$

CRISP3	cysteine−rich secretory protein 3	6p12.3	10321	0,46	3,42	$6{,}79 \times 10^{-04}$	$1{,}96 \times 10^{-03}$
CLDN16	claudin 16	3q28	10686	0,54	3,41	$6{,}95 \times 10^{-04}$	$1{,}98 \times 10^{-03}$
C3	complement component 3	19p13.3−p13.2	718	0,39	3,37	$7{,}94 \times 10^{-04}$	$2{,}18 \times 10^{-03}$
INDO	indoleamine−pyrrole 2,3 dioxygenase	8p12−p11	3620	0,43	3,37	$7{,}99 \times 10^{-04}$	$2{,}19 \times 10^{-03}$
VTCN1	V−set domain containing T cell activation inhibitor 1	1p13.1	79679	0,43	3,37	$8{,}14 \times 10^{-04}$	$2{,}21 \times 10^{-03}$
PART1	prostate androgen−regulated transcript 1	5q12.1	25859	0,39	3,27	$1{,}16 \times 10^{-03}$	$2{,}86 \times 10^{-03}$
MT1G	metallothionein 1G	16q13	4495	0,35	3,08	$2{,}17 \times 10^{-03}$	$4{,}42 \times 10^{-03}$
SPON1	spondin 1, extracellular matrix protein	11p15.2	10418	0,35	3,07	$2{,}26 \times 10^{-03}$	$4{,}55 \times 10^{-03}$
SOSTDC1	sclerostin domain containing 1	7p21.1	25928	0,36	3,06	$2{,}33 \times 10^{-03}$	$4{,}65 \times 10^{-03}$
MT1H	metallothionein 1H	16q13	4496	0,37	2,88	$4{,}17 \times 10^{-03}$	$6{,}98 \times 10^{-03}$
SLC5A1	solute carrier family 5 (sodium/glucose cotransporter), member 1	22q13.1|22q12.3	6523	0,34	2,80	$5{,}23 \times 10^{-03}$	$8{,}12 \times 10^{-03}$
IMPG2	interphotoreceptor matrix proteoglycan 2	3q12.2−q12.3	50939	0,34	2,70	$7{,}12 \times 10^{-03}$	$1{,}00 \times 10^{-02}$
AMY1A	amylase alpha 1A	1p21.1	276	0,36	2,58	$1{,}03 \times 10^{-02}$	$1{,}28 \times 10^{-02}$
PCP4	Purkinje cell protein 4	21q22.2	5121	0,34	2,10	$3{,}60 \times 10^{-02}$	$3{,}17 \times 10^{-02}$
FABP4	fatty acid binding protein 4, adipocyte	8q21	2167	−0,38	−2,61	$9{,}21 \times 10^{-03}$	$1{,}19 \times 10^{-02}$
POSTN	periostin, osteoblast specific factor	13q13.3	10631	−0,52	−2,86	$4{,}38 \times 10^{-03}$	$7{,}24 \times 10^{-03}$
FAP	fibroblast activation protein, alpha	2q23	2191	−0,40	−2,91	$3{,}74 \times 10^{-03}$	$6{,}44 \times 10^{-03}$
SFRP4	secreted frizzled−related protein 4	7p14.1	6424	−0,41	−3,19	$1{,}52 \times 10^{-03}$	$3{,}46 \times 10^{-03}$
LUM	lumican	12q21.3−q22	4060	−0,45	−3,23	$1{,}32 \times 10^{-03}$	$3{,}13 \times 10^{-03}$
CCDC80	coiled−coil domain containing 80	3q13.2	151887	−0,34	−3,26	$1{,}19 \times 10^{-03}$	$2{,}92 \times 10^{-03}$
ACTA2	actin, alpha 2, smooth muscle, aorta	10q23.3	59	−0,33	−3,50	$4{,}93 \times 10^{-04}$	$1{,}56 \times 10^{-03}$
BCAT1	branched chain aminotransferase 1, cytosolic	12p12.1	586	−0,42	−3,52	$4{,}68 \times 10^{-04}$	$1{,}51 \times 10^{-03}$
FN1	fibronectin 1	2q34	2335	−0,38	−3,63	$3{,}06 \times 10^{-04}$	$1{,}11 \times 10^{-03}$
CCND2	cyclin D2	12p13	894	−0,33	−3,64	$2{,}97 \times 10^{-04}$	$1{,}08 \times 10^{-03}$
CPM	carboxypeptidase M	12q14.3	1368	−0,34	−3,71	$2{,}27 \times 10^{-04}$	$8{,}99 \times 10^{-04}$
COL5A2	collagen, type V, alpha 2	2q14−q32	1290	−0,36	−3,73	$2{,}13 \times 10^{-04}$	$8{,}59 \times 10^{-04}$
DSCR8	Down syndrome critical region gene 8	21q22.2	84677	−0,36	−3,81	$1{,}57 \times 10^{-04}$	$6{,}88 \times 10^{-04}$
THBS2	thrombospondin 2	6q27	7058	−0,45	−3,83	$1{,}42 \times 10^{-04}$	$6{,}39 \times 10^{-04}$
COL1A1	collagen, type I, alpha 1	17q21.33	1277	−0,37	−3,84	$1{,}36 \times 10^{-04}$	$6{,}21 \times 10^{-04}$
COL1A2	collagen, type I, alpha 2	7q22.1	1278	−0,35	−3,92	$1{,}00 \times 10^{-04}$	$5{,}00 \times 10^{-04}$
BEX1	brain expressed, X−linked 1	Xq21−q23|Xq22	55859	−0,40	−3,92	$9{,}90 \times 10^{-05}$	$4{,}97 \times 10^{-04}$
TMSL8	thymosin−like 8	Xq21.33−q22.3	11013	−0,46	−3,93	$9{,}61 \times 10^{-05}$	$4{,}86 \times 10^{-04}$
GJA1	gap junction protein, alpha 1, 43kDa	6q21−q23.2	2697	−0,37	−4,12	$4{,}29 \times 10^{-05}$	$2{,}67 \times 10^{-04}$

COL3A1	collagen, type III, alpha 1	2q31	1281	−0,46	−4,19	$3,22 \times 10^{-05}$	$2,15 \times 10^{-04}$
SLC6A15	solute carrier family 6 (neutral amino acid transporter), member 15	12q21.3	55117	−0,41	−4,35	$1,60 \times 10^{-05}$	$1,27 \times 10^{-04}$
FBN1	fibrillin 1	15q21.1	2200	−0,43	−4,40	$1,27 \times 10^{-05}$	$1,08 \times 10^{-04}$
COL6A3	collagen, type VI, alpha 3	2q37	1293	−0,41	−4,41	$1,24 \times 10^{-05}$	$1,06 \times 10^{-04}$
TMEFF1	transmembrane protein with EGF−like and two follistatin−like domains 1	9q31	8577	−0,41	−4,45	$1,02 \times 10^{-05}$	$9,23 \times 10^{-05}$
UCHL1	ubiquitin carboxyl−terminal esterase L1 (ubiquitin thiolesterase)	4p14	7345	−0,51	−4,46	$9,95 \times 10^{-06}$	$9,08 \times 10^{-05}$
HIST1H4L	histone cluster 1, H4l	6p22−p21.3	8368	−0,38	−4,47	$9,53 \times 10^{-06}$	$8,82 \times 10^{-05}$
TMEM56	transmembrane protein 56	1p21.3	148534	−0,35	−4,51	$7,91 \times 10^{-06}$	$7,61 \times 10^{-05}$
DDR2	discoidin domain receptor tyrosine kinase 2	1q23.3	4921	−0,33	−4,54	$6,88 \times 10^{-06}$	$6,85 \times 10^{-05}$
ALDH1A2	aldehyde dehydrogenase 1 family, member A2	15q22.1	8854	−0,44	−4,64	$4,31 \times 10^{-06}$	$4,81 \times 10^{-05}$
GPC3	glypican 3	Xq26.1	2719	−0,45	−4,67	$3,80 \times 10^{-06}$	$4,37 \times 10^{-05}$
CENPF	centromere protein F, 350/400ka (mitosin)	1q32−q41	1063	−0,32	−4,69	$3,38 \times 10^{-06}$	$4,01 \times 10^{-05}$
BICC1	BicC family RNA binding protein 1	10q21.1	80114	−0,40	−4,75	$2,57 \times 10^{-06}$	$3,27 \times 10^{-05}$
IGF2BP3	insulin−like growth factor 2 mRNA binding protein 3	7p11	10643	−0,62	−4,78	$2,27 \times 10^{-06}$	$2,96 \times 10^{-05}$
FREM2	FRAS1 related extracellular matrix 2	13q13.3	341640	−0,33	−4,86	$1,52 \times 10^{-06}$	$2,17 \times 10^{-05}$
LTBP1	latent transforming growth factor beta binding protein 1	2p22−p21	4052	−0,34	−4,87	$1,44 \times 10^{-06}$	$2,06 \times 10^{-05}$
CDH11	cadherin 11, type 2, OB−cadherin (osteoblast)	16q22.1	1009	−0,46	−4,94	$1,04 \times 10^{-06}$	$1,60 \times 10^{-05}$
SEMA3D	sema domain, immunoglobulin domain (Ig), short basic domain, secreted, (semaphorin) 3D	7q21.11	223117	−0,40	−4,96	$9,46 \times 10^{-07}$	$1,50 \times 10^{-05}$
PLCB4	phospholipase C, beta 4	20p12	5332	−0,35	−4,96	$9,25 \times 10^{-07}$	$1,48 \times 10^{-05}$
VCAN	versican	5q14.3	1462	−0,56	−4,99	$7,98 \times 10^{-07}$	$1,34 \times 10^{-05}$
MYO1B	myosin IB	2q12−q34	4430	−0,37	−5,10	$4,55 \times 10^{-07}$	$8,66 \times 10^{-06}$
PLAT	plasminogen activator, tissue	8p12	5327	−0,45	−5,15	$3,64 \times 10^{-07}$	$7,29 \times 10^{-06}$
ERRFI1	ERBB receptor feedback inhibitor 1	1p36	54206	−0,33	−5,19	$2,88 \times 10^{-07}$	$5,96 \times 10^{-06}$
LAMB1	laminin, beta 1	7q22	3912	−0,39	−5,57	$3,89 \times 10^{-08}$	$1,28 \times 10^{-06}$
LEF1	lymphoid enhancer−binding factor 1	4q23−q25	51176	−0,44	−5,59	$3,53 \times 10^{-08}$	$1,20 \times 10^{-06}$
DPYSL3	dihydropyrimidinase−like 3	5q32	1809	−0,37	−5,66	$2,40 \times 10^{-08}$	$8,79 \times 10^{-07}$
FSTL1	follistatin−like 1	3q13.33	11167	−0,39	−5,71	$1,82 \times 10^{-08}$	$6,97 \times 10^{-07}$
TOP2A	topoisomerase (DNA) II alpha 170kDa	17q21−q22	7153	−0,42	−5,74	$1,58 \times 10^{-08}$	$6,37 \times 10^{-07}$
ANTXR1	anthrax toxin receptor 1	2p13.1	84168	−0,37	−5,88	$6,83 \times 10^{-09}$	$3,34 \times 10^{-07}$
MPDZ	multiple PDZ domain protein	9p24−p22	8777	−0,35	−5,97	$4,08 \times 10^{-09}$	$2,28 \times 10^{-07}$

FYN	FYN oncogene related to SRC, FGR, YES	6q21	2534	−0,36	−6,10	$1{,}97 \times 10^{-09}$	$1{,}30 \times 10^{-07}$
PTPN13	protein tyrosine phosphatase, non−receptor type 13 (APO−1/CD95 (Fas)−associated phosphatase)	4q21.3	5783	−0,66	−6,16	$1{,}35 \times 10^{-09}$	$9{,}95 \times 10^{-08}$
F2R	coagulation factor II (thrombin) receptor	5q13	2149	−0,40	−6,19	$1{,}16 \times 10^{-09}$	$8{,}96 \times 10^{-08}$
CENPO	centromere protein O	2p23.3	79172	−0,33	−6,61	$8{,}52 \times 10^{-11}$	$1{,}02 \times 10^{-08}$
MCOLN3	mucolipin 3	1p22.3	55283	−0,44	−6,89	$1{,}51 \times 10^{-11}$	$2{,}51 \times 10^{-09}$
DZIP1	DAZ interacting protein 1	13q32.1	22873	−0,41	−6,89	$1{,}50 \times 10^{-11}$	$2{,}51 \times 10^{-09}$
RHOBTB3	Rho−related BTB domain containing 3	5q15	22836	−0,41	−6,94	$1{,}05 \times 10^{-11}$	$1{,}94 \times 10^{-09}$
FGD6	FYVE, RhoGEF and PH domain containing 6	12q22	55785	−0,36	−7,47	$3{,}09 \times 10^{-13}$	$1{,}63 \times 10^{-10}$
PRKD3	protein kinase D3	2p21	23683	−0,37	−8,01	$6{,}48 \times 10^{-15}$	$6{,}81 \times 10^{-12}$
SKIL	SKI−like oncogene	3q26	6498	−0,51	−8,03	$5{,}65 \times 10^{-15}$	$6{,}81 \times 10^{-12}$

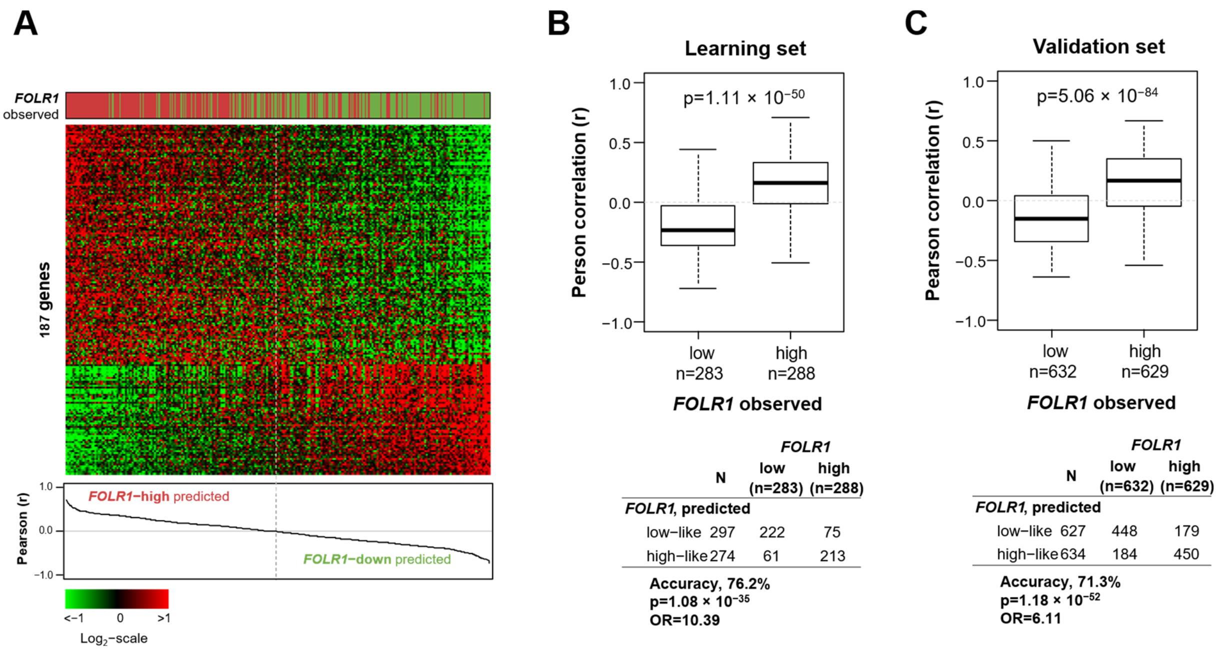

Supplementary Figure S4: Validation of the 187 genes differentially expressed between the FOLR1 expression classes

(A) Heatmap of the 187 genes in the learning set (TCGA 571 OC samples). Samples were sorted by decreasing Pearson correlation coefficient with the FOLR1 profile (bottom panel) and the 187 genes were sorted by statistical test from the FOLR1 supervised analysis. FOLR1 expression classes are color−coded in red and green for High and low group, respectively (top panel). (B) Box plot of Pearson coefficient of expression profile of each sample in the learning set (TCGA) according to FORL1 mRNA classes, significance assessed by using t−test (top panel); and, contingency table of observed and predicted FOLR1 classes at binary level, significance assessed by using Fisher's Exact test (bottom panel). (C) same as (B) but in the independent validation set.

A

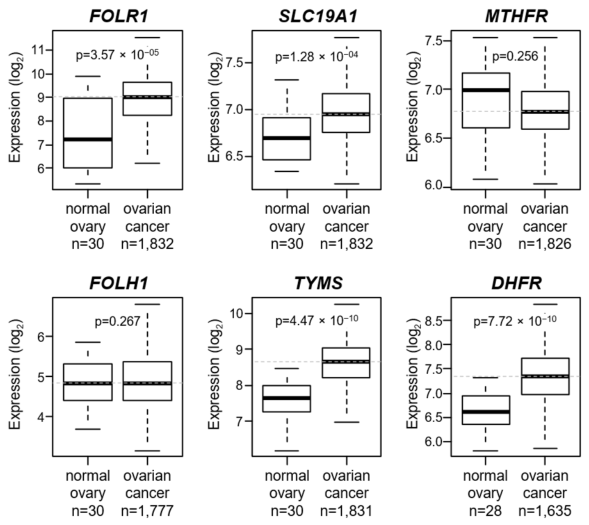

B

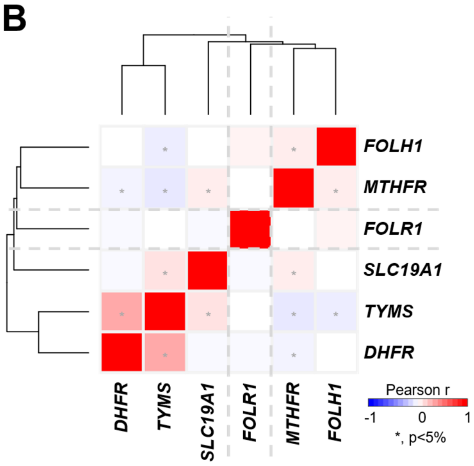

C

OS	N	Univariate HR [95%CI]	p-value
FOLR1, high vs. low	1,471	0.93 [0.81-1.07]	0.326
SLC19A1, high vs. low	1,472	0.84 [0.73-0.96]	1.11×10^{-02}
MTHFR, high vs. low	1,472	1.05 [0.91-1.20]	0.525
FOLH1, high vs. low	1,471	1.07 [0.93-1.22]	0.353
TYMS, high vs. low	1,471	0.98 [0.85-1.12]	0.768
DHFR, high vs. low	1,283	1.02 [0.88-1.19]	0.767

D

PFS	N	Univariate HR [95%CI]	p-value
FOLR1, high vs. low	1,207	0.99 [0.86-1.14]	0.918
SLC19A1, high vs. low	1,208	0.92 [0.80-1.05]	0.214
MTHFR, high vs. low	1,208	1.01 [0.88-1.16]	0.920
FOLH1, high vs. low	1,207	1.03 [0.90-1.19]	0.638
TYMS, high vs. low	1,207	0.98 [0.85-1.12]	0.733
DHFR, high vs. low	1,176	0.97 [0.85-1.12]	0.719

Supplementary Figure S5: mRNA expression of members of the folate signaling pathway

(A) Box plot of mRNA expression according type of sample, significance assessed by Student’s t-test. (B) Correlation matrix heatmap of folate signaling pathway genes across ovarian cancer samples, significance assessed by using Pearson’s correlation test. (C) Univariate Cox regression analysis of overall survival according the folate signaling pathway genes, significance assessed by Wald’s test. (D) Univariate Cox regression analysis of progression−free survival according the folate signaling pathway genes, significance assessed by Wald’s test.

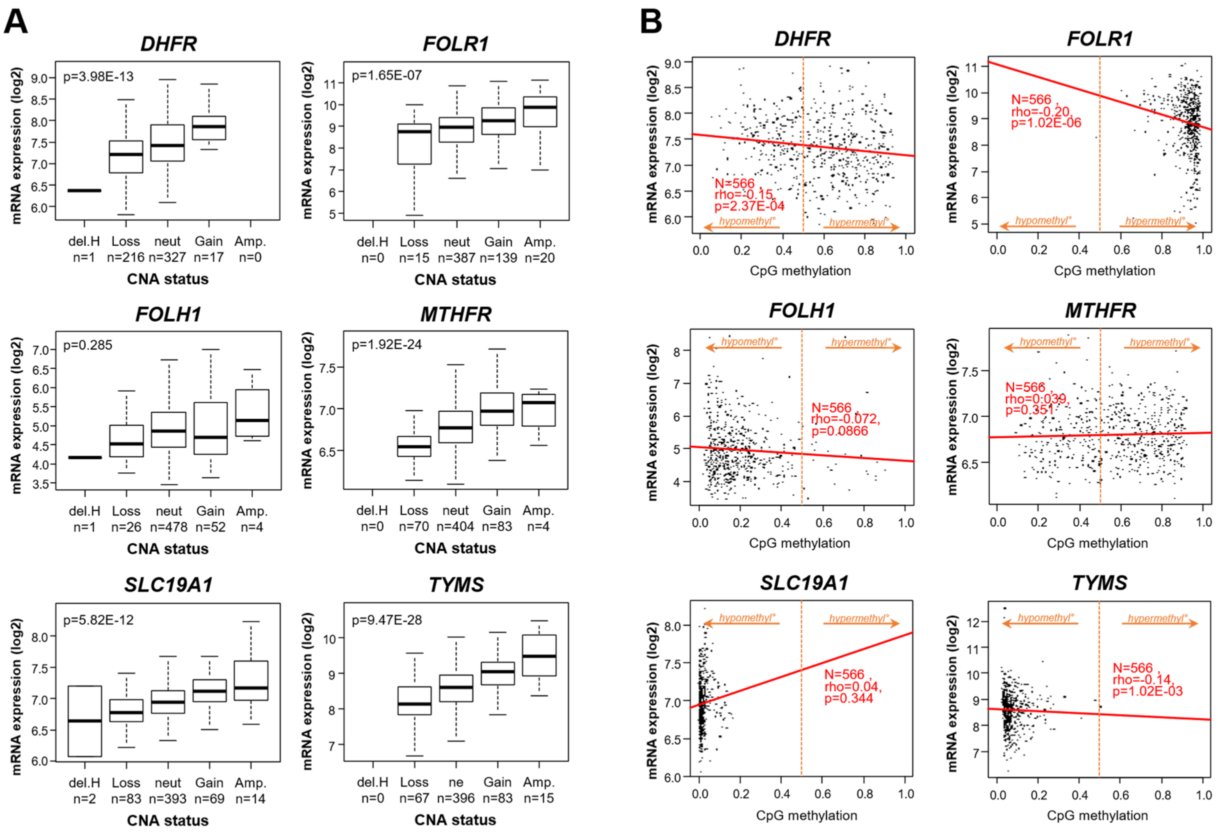

Supplementary Figure S6 : Correlation of mRNA expression of FOLR1 and other genes involved in the folate signaling pathway with (A) gene copy number alterations, by using box plot and one−way ANOVA test, and (B) promoter DNA methylation by using scatterplot of mRNA expression and CpG methylation values and Spearman correlation test.

Abbreviations : del.H, homozygous deletion (CN=−2); Loss, heterozygous loss (CN=−1); neut, neutral; Gain, one−copy gain (CN=+1); Amp., amplification (CN>=+2)
